# Type I interferon autoantibodies in hospitalized patients with Middle East respiratory syndrome and association with outcomes and treatment effect of interferon beta‐1b in MIRACLE clinical trial

**DOI:** 10.1111/irv.13116

**Published:** 2023-03-21

**Authors:** Faizah Alotaibi, Naif Khalaf Alharbi, Lindsey B. Rosen, Ayed Y. Asiri, Abdullah M. Assiri, Hanan H. Balkhy, Majed Al Jeraisy, Yasser Mandourah, Sameera AlJohani, Shmeylan Al Harbi, Hani A. Aziz Jokhdar, Ahmad M. Deeb, Ziad A. Memish, Jesna Jose, Sameeh Ghazal, Sarah Al Faraj, Ghaleb A. Al Mekhlafi, Nisreen Murad Sherbeeni, Fatehi Elnour Elzein, Badriah M. AlMutairi, Abdulaziz Al‐Dawood, Mashan L. Abdullah, Tlili Barhoumi, Mohammed W. Alenazi, Abdulrahman Almasood, Steven M. Holland, Yaseen M. Arabi, the Saudi Critical Care Trials Group

**Affiliations:** ^1^ College of Science and Health Professions King Saud bin Abdulaziz University for Health Sciences and King Abdullah International Medical Research Center, Ministry of National Guard Health Affairs Riyadh Saudi Arabia; ^2^ King Saud bin Abdulaziz University for Health Sciences and King Abdullah International Medical Research Center Riyadh Saudi Arabia; ^3^ Laboratory of Clinical Immunology and Microbiology, Division of Intramural Research National Institute of Allergy and Infectious Diseases (NIAID), National Institutes of Health (NIH) MD Bethesda USA; ^4^ Prince Mohammed bin Abdulaziz Hospital Riyadh Saudi Arabia; ^5^ Ministry of Health Riyadh Saudi Arabia; ^6^ Antimicrobial Resistance Division World Health Organization Geneva Switzerland; ^7^ Prince Sultan Military Medical City Riyadh Saudi Arabia; ^8^ Department of Pathology and Laboratory Medicine King Abdulaziz Medical City, Ministry of National Guard Health Affairs Riyadh Saudi Arabia; ^9^ Pharmaceutical Care Department King Abdulaziz Medical City, Ministry of National Guard Health Affairs Riyadh Saudi Arabia; ^10^ Prince Mohammed bin Abdulaziz Hospital, Ministry of Health, College of Medicine Alfaisal University, Riyadh, Kingdom of Saudi Arabia, Hubert Department of Global Health, Rollins School of Public Health, Emory University Georgia Atlanta USA; ^11^ Experimental Medicine Department, King Abdullah International Medical Research Center King Saud bin Abdulaziz University for Health Sciences Riyadh Saudi Arabia; ^12^ Intensive Care Department King Abdulaziz Medical City, Ministry of National Guard Health Affairs Riyadh Saudi Arabia

**Keywords:** auto‐abs, ICU, MERS, MIRACLE trial, Type I IFNs

## Abstract

**Background:**

Type I interferons (IFNs) are essential antiviral cytokines induced upon respiratory exposure to coronaviruses. Defects in type I IFN signaling can result in severe disease upon exposure to respiratory viral infection and are associated with worse clinical outcomes. Neutralizing autoantibodies (auto‐Abs) to type I IFNs were reported as a risk factor for life‐threatening COVID‐19, but their presence has not been evaluated in patients with severe Middle East respiratory syndrome (MERS).

**Methods:**

We evaluated the prevalence of type I IFN auto‐Abs in a cohort of hospitalized patients with MERS who were enrolled in a placebo‐controlled clinical trial for treatment with IFN‐β1b and lopinavir‐ritonavir (MIRACLE trial). Samples were tested for type I IFN auto‐Abs using a multiplex particle‐based assay.

**Results:**

Among the 62 enrolled patients, 15 (24.2%) were positive for immunoglobulin G auto‐Abs for at least one subtype of type I IFNs. Auto‐Abs positive patients were not different from auto‐Abs negative patients in age, sex, or comorbidities. However, the majority (93.3%) of patients who were auto‐Abs positive were critically ill and admitted to the ICU at the time of enrollment compared to 66% in the auto‐Abs negative patients. The effect of treatment with IFN‐β1b and lopinavir‐ritonavir did not significantly differ between the two groups.

**Conclusion:**

This study demonstrates the presence of type I IFN auto‐Abs in hospitalized patients with MERS.

## 1 INTRODUCTION

The Middle East respiratory syndrome (MERS) was first reported in Saudi Arabia in 2012, and as of January 2023, it has caused 2603 cases and 935 associated deaths.^1^ MERS is caused by the MERS coronavirus (MERS‐CoV), a virus that belongs to the coronavirus family, which includes also SARS‐CoV‐1 and SARS‐CoV‐2, which resulted in major outbreaks over the past two decades known as severe acute respiratory syndrome (SARS) and coronavirus disease 19 (COVID‐19), respectively.^2^ The clinical presentation of MERS ranges from asymptomatic infection to severe pneumonia and multi‐organ failure^3^ ^,^ ^4^ with a case fatality rate of 34.3%, which is higher than both SARS and COVID‐19.^1^, ^5^, ^6^ A decade after its first identification, MERS cases continue to occur sporadically and are considered a public health concern.

Type I interferons (IFNs) are essential antiviral cytokines induced upon human exposure to respiratory viruses such as MERS‐CoV.^7^ It has been reported that inborn errors of IFN involving regulatory factor 7 (IRF7)–dependent type I IFN induction and Toll‐like receptor 3 (TLR3) are associated with life‐threatening COVID‐19 pneumonia in a small subset of patients.^8^ Furthermore, autoantibodies (auto‐Abs) against IFN‐α2 and/or IFN‐ω were reported in at least 10% of patients with life‐threatening COVID‐19 pneumonia but not in individuals with asymptomatic or mild infection.^9^ These auto‐Abs detected in serum and plasma can neutralize IFN‐α2 and reduce or eliminate type I IFN responses.^9^ These observations were later confirmed in independent cohort studies from different countries.^10^, ^11^, ^12^, ^13^ In addition, approximately 0.3% of general population samples collected before the pandemic were positive for at least one type of type I IFN auto‐Abs,^9^ with a sharp increase with age,^14^ which suggests that these auto‐Abs were not solely triggered by viral infection. The existence of these auto‐Abs might be genetically driven because they were found in patients with autoimmune polyendocrine syndrome type‐1 (APS‐1) caused by autoimmune regulator (AIRE) germline mutations.^15^, ^16^ Of note, patients with APS‐1 have been shown to have a higher risk of developing severe or critical COVID‐19 pneumonia.^17^ Production of these auto‐Abs is also seen in patients with a combination of immunodeficiency and hypomorphic mutations of RAG1 or RAG2,^18^ thymoma,^19^ systemic lupus erythematosus,^20^ and myasthenia gravis.^21^ However, the clinical implications of these auto‐Abs in these diseases are not well understood. Auto‐Abs in patients with MERS have not been studied before.

In this study, we evaluated the presence of type I IFN auto‐Abs, including IFN‐α2, β, and/or IFN‐ω in hospitalized patients with MERS; examined their association with mortality and other clinical outcomes; and evaluated whether the presence of type I IFN auto‐Abs affected the response to treatment with IFN beta‐1b and lopinavir‐ritonavir.

## 2 MATERIALS AND METHODS

### 2.1 The study design

This is a follow‐up study of the MIRACLE trial (ClinicalTrials.gov number NCT02845843).^22^, ^23^, ^24^ This was an adaptive, randomized, double‐blind, placebo‐controlled trial that evaluated the efficacy of recombinant IFN‐β1b and lopinavir‐ritonavir compared with placebo, on 90‐day all‐cause mortality in hospitalized patients with laboratory‐confirmed MERS.^22^, ^23^, ^24^ The study (*n* = 95) found that recombinant IFN‐β1b and lopinavir‐ritonavir resulted in lower 90‐day mortality in hospitalized patients with laboratory‐confirmed MERS. The study was sponsored by King Abdullah International Medical Research Center, Riyadh, Saudi Arabia. Details of the study and its findings have already been published.^22^, ^23^, ^24^ The main trial, including this current study, was approved by the Institutional Review Board at the participating sites, and informed consent was obtained for participation in the main study and this current study.

### 2.2 Subjects and samples

Blood samples were collected from enrolled patients from three recruiting sites in Riyadh, Saudi Arabia, between November 2016 and April 2020. MERS infection was confirmed by real‐time reverse transcriptase–polymerase chain reaction (RT‐PCR) assay. For this study, we used plasma samples that were collected in heparin tubes on Study Day 1, before the administration of study drugs.

### 2.3 Detection of auto‐abs against type I IFNs

Plasma samples from patients with MERS were tested at King Abdullah International Research Center laboratory, Riyadh, Saudi Arabia, for auto‐Abs against IFN‐α2, β, and IFN‐ω using a multiplex particle‐based assay that uses magnetic beads with differential fluorescence that covalently coupled to recombinant human proteins (2.5 μg/reaction) as provided by the National Institutes of Health (NIH), Bethesda, Maryland, United States.^9^ As previously described,^9^ coupled beads were incubated with plasma samples in 1:100 dilution for 30 min. Following incubation, beads were washed and stained with PE‐labeled goat anti‐human IgG (1 μg/ml) for 1 h. Beads were then washed again, and the result was acquired using a BioPlex 3D instrument in a multiplex assay. Confirmed positive and negative samples that are provided by the NIH^9^ along with technical controls from King Abdullah International Research Center were used to determine a threshold. Samples with fluorescence intensity (FI) of >800 for IFN‐α2 and IFN‐ω or >600 for IFN‐β were considered positive for auto‐Abs and were tested for blocking activity.

### 2.4 MERS pseudotyped viral particles (MERSpp) neutralization assay

Although type I IFNs are thought to be beneficial against viral infections, only IFN‐α2 controls early viral dissemination and prevents virus entry into the cell at the early stage of infection.^25^ Therefore, samples from patients with MERS who tested positive for IFN‐α2 auto‐Abs (*n* = 9) and samples from patients who tested negative for type I IFNs' auto‐Abs (*n* = 4) were further evaluated by neutralization assay to examine IFN‐α2 auto‐Abs neutralizing activity to block IFN‐α2 antiviral function in vitro. MERSpp were produced and titrated at King Abdullah International Research Center laboratory, Riyadh, Saudi Arabia, using human embryonic kidney (HEK) 293T cells as described previously.^26^ We examined samples with the IFN‐α2 auto‐Abs and found samples with auto‐Abs highly neutralizing the ability of IFN‐α2 to block the infection of MERSpp in human hepatoma 7.5 (Huh7.5) cells. Huh7.5 cells were cultured with Dulbecco's Modified Eagle Medium (DMEM) supplemented 10% fetal bovine serum (FBS) at 37°C and 5% CO_2_ condition in a 96‐well plate at a density of 10 × 10^3^ cells/well. The next day, patient plasma samples were prepared in a 1:100 dilution and incubated with a standard concentration of IFN‐α2 (30 ng/ml) for 1 h at 37°C before transferring the mixture to the cells. Following the incubation, Huh7.5 cells were washed and stimulated with the mixture for 16 h before incubation with MERSpp at 12.5 of multiplicity of infection (MOI). After 48 h incubation period at 37°C in 5% CO_2_, cells were fixed with 7% formaldehyde and then lysed, and the assay was developed using the Bright‐Glo™ Luciferase Assay System (Promega, Madison, WIS, USA), and luciferase activity was measured using a luminometer. Cells only and cells treated with MERSpp only were used as a control to determine 100% and 0% neutralization activity. The percentage of infected cells was calculated as the following:

$$\%\text{of infected cells}=\frac{\text{Cells}+\text{virus}+\text{Plasma}+\text{Type}\;I\;\text{IFNs}}{\text{Cells}+\text{Virus only}}\times 100$$

### 2.5 Statistical analysis

Continuous data are presented as medians and interquartile ranges. Categorical data are presented as numbers and percentages. Statistical differences between samples were assessed using chi‐square, Fisher's exact test, Student's t‑test, or Mann–Whitney test as appropriate. Analyses were performed using SAS 9.4 (SAS Institute, Cary, NC) and GraphPad Prism 8.2.1 (GraphPad, San Diego, CA). Statistical tests for variables were performed using a two‐sided alpha value of 0.05 to denote a significant level.

## 3 RESULTS

### 3.1 Auto‐abs against IFN‐α2, IFN‐β, and/or IFN‐ω in hospitalized patients with MERS

**FIGURE 1:**
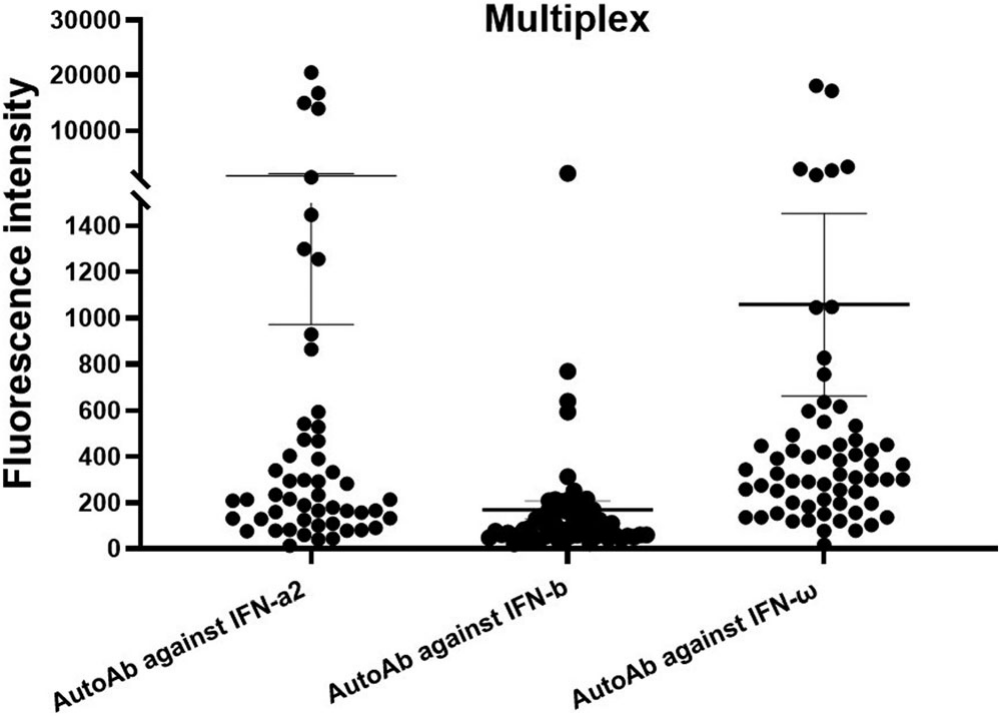
Multiplex particle‐based assay for autoantibodies (auto‐Abs) against interferon (IFN)‐α2, IFN‐β, and IFN‐ω in hospitalized patients with Middle East respiratory syndrome (MERS) (*n* = 62). Samples with fluorescence intensity (FI) of >800 for IFN‐α2 and IFN‐ω or >600 for IFN‐β were considered positive for auto‐Abs. Data are mean ± SEM.

**FIGURE 2:**
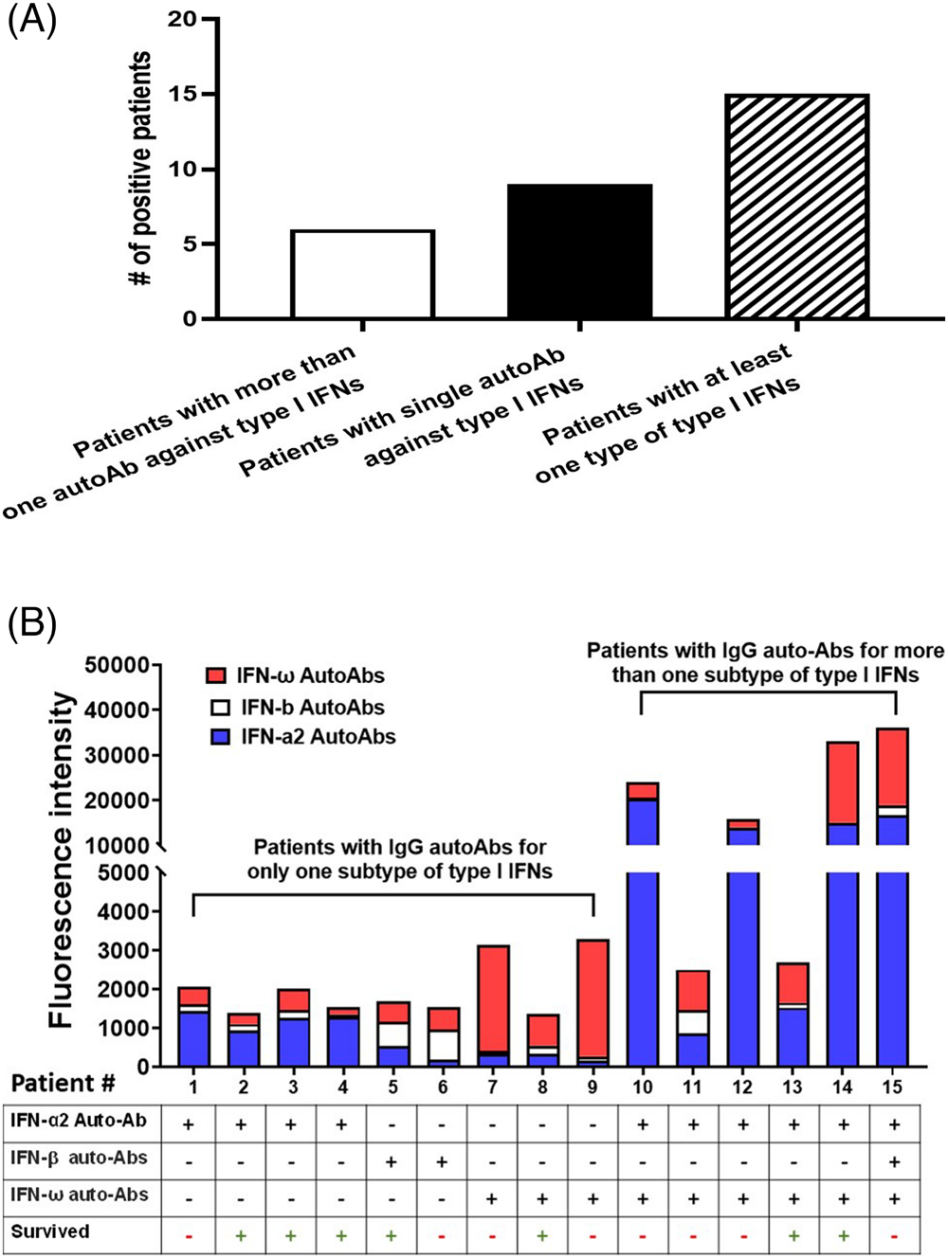
(A) Distribution of the number of positive samples for patients with immunoglobulin G autoantibodies (auto‐Abs) for more than one subtype of type I interferons (IFNs) (IFN‐α2, IFN‐ω, and IFN‐β) and (IFN‐α2 and IFN‐ω) and patients with immunoglobulin G auto‐Abs for only one subtype of type I IFNs. (B) Distribution of auto‐Ab titers of patients with positive auto‐Abs samples and mortality.

Among the 62 enrolled patients with laboratory‐confirmed MERS, 15 patients (24.2%) were positive for immunoglobulin G auto‐Abs for at least one subtype of type I IFNs, including IFN‐α2, IFN‐β, and/or IFN‐ω (Figure 1). Among the 15 patients, six patients had immunoglobulin G auto‐Abs for more than one subtype of type I IFNs (IFN‐α2, IFN‐β, and IFN‐ω) or (IFN‐α2 and IFN‐ω), whereas nine patients had immunoglobulin G auto‐Abs for only one subtype of type I IFNs (Figure 2A). The titers for auto‐Abs against IFN‐α2, IFN‐β, and IFN‐ω are shown in (Figure 2B). Notably, four out of six patients with immunoglobulin G auto‐Abs for more than one subtype of type I IFNs died. In contrast, four out of nine patients with immunoglobulin G auto‐Abs for only one subtype of type I IFNs died (Figure 1B).

### 3.2 The auto‐abs neutralization IFN‐α2 in vitro

**FIGURE 3:**
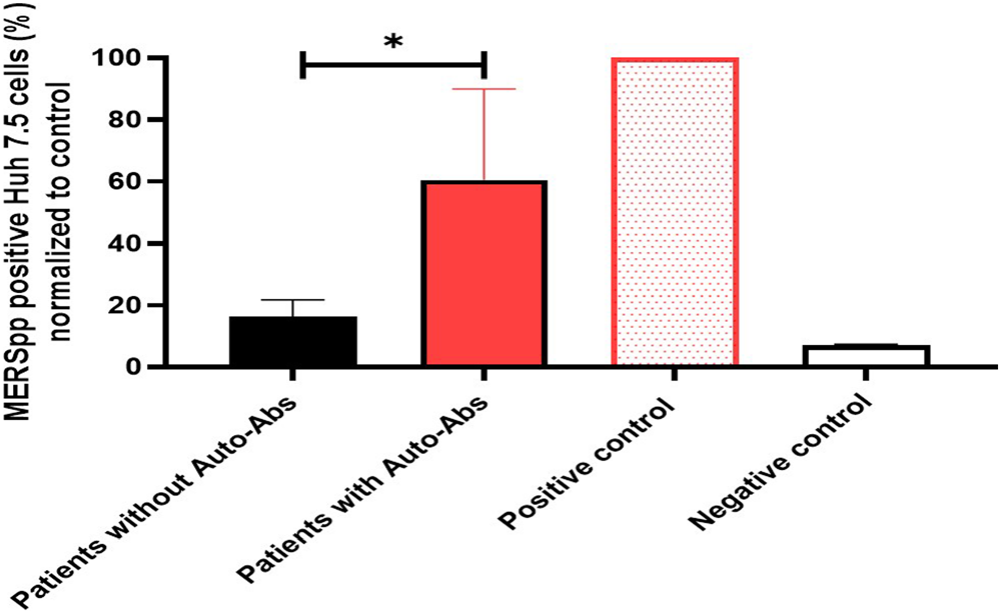
Increased MERS pseudotyped viral particles' (MERSpp) infection, despite the presence of interferon (IFN)‐α2, in the presence of plasma with autoantibodies (auto‐Abs) targeting IFN‐α2. MERSpp infection measured 48 h after infection in Huh7.5 cells treated with IFN‐α2 in the presence of plasma from patients with auto‐Abs or without auto‐Abs. Samples from patients with positive auto‐Abs for IFN‐α2 (*n* = 9) and samples from patients with negative auto‐Abs for type I IFNs (*n* = 4). Asterisks represent statistical significance *p* ≤ 0.05 (Mann–Whitney test).

Overall, samples from patients with auto‐Abs against IFN‐α2 have neutralizing activity to IFN‐α2 and limit the antiviral activity of IFN‐α2 against MERSpp (Figure 3). In contrast, samples from patients without auto‐Abs did not block IFN‐α2 antiviral activity against MERSpp (Figure 3). Notably, five out of six (83.3%) with highly neutralizing Auto‐Abs died compared to one out of three (33.3%) in patients with low neutralizing activity in vitro (Supporting information Table S1).

### 3.3 Clinical characteristics of patients with or without auto‐Abs for type I IFNs

In this study, the median age of the auto‐Abs positive patients was 56 years and 60 years for the auto‐Abs negative patients, and males represented 80% and 83%, respectively (Table 1A). Comorbidities during the disease course were similar between the two groups, including chronic cardiac disease, chronic pulmonary disease, chronic renal disease, and diabetes with chronic complications (Table 1A). The majority (93.3%) of patients who were auto‐Abs positive were critically ill and admitted to the ICU at the time of enrollment compared to 66% in the auto‐Abs negative patients, (*p* = 0.049) (Table 1A). Interventions before randomization including renal replacement therapy, vasopressor therapy, and the use of corticosteroids were also similar between the two groups (Table 1A).

**TABLE 1:** (A) Baseline characteristics of patients in the trial and (B) study interventions and co‐interventions during the trial period.

A. Baseline characteristics of patients in the trial			
Variable	Autoantibodies positive (*N* = 15)	Autoantibodies negative (*N* = 47)	*p* value
Age (years)—median (IQR)	56 (44, 69)	60 (47, 68)	0.86*
Male sex—no. (%)	12 (80.0)	39 (83.0)	>0.99^^
Body mass index (kg/m^2^)—mean ± SD	28.4 ± 5.66	27.1 ± 5.90	0.46*
APACHE II^‡^ − mean ± SD	24.1 ± 7.14	20.8 ± 10.53	0.26*
SOFA score, median (IQR)	8.0 (7.0, 13.0)	6.0 (4.0, 9.0)	0.11^
Karnofsky performance status score—median (IQR)	90.0 (70.0, 100.0)	90.0 (70.0, 100.0)	0.97^
**Comorbidities—no. (%)**			
Any chronic comorbidity	13 (86.7)	39 (83.0)	>0.99^^
Chronic cardiac disease	5 (33.3)	9 (19.1)	0.30^^
Chronic pulmonary disease	1 (6.7)	1 (2.1)	0.43^^
Chronic renal disease	2 (13.3)	16 (34.0)	0.19^^
Diabetes with chronic complications	9 (60.0)	15 (31.9)	0.0519**
**Location at time of randomization—no. (%)**			
Ward	1 (6.7)	16 (34.0)	0.049^^
ICU	14 (93.3)	31 (66.0)	
**Randomization stratum—no. (%)**			
Mechanically ventilated	10 (66.7)	19 (40.4)	0.08**
Not mechanically ventilated	5 (33.3)	28 (59.6)	
**Interventions before randomization—no. (%)**			
Renal replacement therapy	3 (20.0)	18 (38.3)	0.19**
Vasopressor therapy	5 (33.3)	8 (17.0)	0.27^^
Corticosteroids	8 (53.3)	15 (31.9)	0.13**

B. Study interventions and co‐interventions during the trial period			
Time of onset of symptoms to randomization, day, and median (IQR)	7.0 (3.0, 11.0)	8.0 (5.0, 12.0)	0.33^
Time of admission to randomization, day, and median (IQR)	2.0 (1.0, 4.0)	2.0 (1.0, 3.0)	0.64^
Number of lopinavir‐ritonavir/placebo doses and median (IQR)	28.0 (18.0, 28.0)	24.0 (11.0, 28.0)	0.08^
Number of interferon‐β1b/placebo injection doses and median (IQR)	7.0 (7.0, 7.0)	6.0 (4.0, 7.0)	0.04^

*Note*: *Plus–minus values are means ± SD. Continuous variables were compared between the two trial groups with the use of an independent *Student's t‑test or ^Mann–Whitney test, and categorical variables were compared with the use of a **chi‑square test or ^^Fisher's exact test. APACHE, acute physiology and chronic health evaluation; IQR, interquartile range; SOFA, sequential organ failure assessment.

* Student's t‑test.

^ Mann–Whitney test.

** Chi‑square test.

^^ Fisher's exact test.

Patients without type I IFN auto‐Abs showed similar favorable responses to treatment interventions compared to patients with type I IFN auto‐Abs as shown in the forest plot (Supporting information Figure S1). The mortality rate was similar between the two groups at all time points (At 28 days, 90 days, and during hospital and/or ICU stay) (Table 2). The overall survival between the two groups was similar at 90‐day follow‐up (Supporting information Figure S2). The median days for virological clearance were similar between the two groups, and both groups had similar Karnofsky performance status at 90 days (Table 2).

**TABLE 2:** Outcomes in patients enrolled in the trial.

Characteristics	Autoantibodies positive (*N* = 15)	Autoantibodies negative (*N* = 47)	*p* value
**Death from any cause—no./total no. (%)**		
Death by Day 90	9/15 (60.0)	18/47 (38.3)	0.14
At 28 days	6/15 (40.0)	14/47 (29.8)	0.46
During ICU stay	7/15 (46.7)	19/47 (40.4)	0.67
During hospital stay	8/15 (53.3)	20/47 (42.6)	0.47
Alive and on renal replacement therapy at Day 90, no. (%)	0/15 (0)	8/47 (17.0)	0.09
Alive and on invasive mechanical ventilation at Day 90, no. (%)	1/15 (6.7)	2/47 (4.3)	0.70
Median no. of days free from invasive or noninvasive mechanical ventilation (IQR)^†^	1.0 (0.0, 19.0)	10.0 (0.0, 28.0)	0.11
Median no. of days free from renal replacement therapy (IQR)^†^	16.0 (0.0, 28.0)	17.0 (0.0, 28.0)	0.94
Median no. of days free from vasopressors (IQR)^†^	19.0 (0.0, 28.0)	26.0 (0.0, 28.0)	0.30
Median no. of days free from organ support (IQR)^†^	0.0 (0.0, 15.0)	9.0 (0.0, 27.0)	0.18
Median no. of days outside the ICU (IQR)^†^	0.0 (0.0, 11.0)	5.0 (0.0, 24.0)	0.057
Hospital length of stay and median (IQR)	25 (14, 41)	16 (8, 40)	0.26
Hospital length of stay among survivors and median (IQR)	27 (20, 59)	18 (12, 45)	0.15
**Virologic outcomes**		
Median days to MERS‐CoV RNA clearance (IQR)^‡^	18.0 (13.0, 22.0)	18.0 (10.0, 26.0)	0.91
Median days to MERS‐CoV RNA clearance among 90‐d survivors (IQR)^‡^	15.5 (10.0, 22.0)	11.0 (9.0, 20.0)	0.90
**Functional outcome**			
Karnofsky performance status score at Day 90 and median (IQR)^§^	0.0 (0.0, 70.0)	50.0 (0.0, 100.0)	0.16

*Note*: Continuous variables were compared between the two groups with the use of Mann–Whitney test, and categorical variables were compared with the use of a chi‑square test or Fisher's exact test.

Abbreviation: MERS‐CoV, MERS coronavirus.

^†^ Calculations of days free from supplemental oxygen, renal replacement therapy, mechanical ventilation, vasopressors, extracorporeal membrane oxygenation, organ support, and days outside the ICU were based on 28 days of observation.

^‡^ Days to MERS‐CoV RNA clearance censored by death or hospital discharge.

^§^ Data on Karnofsky performance status score at Day 90 were not available for one patient in the placebo group. Otherwise, there were no missing values in the variables in this table.

## 4 DISCUSSION

In this study, we found that 24.2% of hospitalized patients with MERS were positive for auto‐Abs against at least one type I IFN. Although patients with auto‐Abs were more likely to be critically ill, the presence of auto‐Abs against at least one type I IFN was not associated with different clinical outcomes or with a difference in the response to treatment with IFN‐β1b and lopinavir‐ritonavir. These observations are similar to those of Abers et al.*,* ^27^ who found increased rates of ICU hospitalization but no increase in mortality with type I IFN auto‐Abs. Our study is the first to investigate the presence of auto‐Abs against IFN‐α2 and IFN‐ω in hospitalized patients with laboratory‐confirmed MERS. Our results are consistent with a recent finding in larger samples of patients with COVID‐19 pneumonia.^9^ Preexisting auto‐Abs were previously reported in a healthy population^9^ and in patients with an autoimmune condition.^17^ This indicates that these auto‐Abs were not triggered solely by MERS or COVID‐19 infections, and their dynamic level could decline rapidly after recovery.^28^

The neutralizing activity of these type I IFN auto‐Abs was also tested using a neutralizing assay in vitro, which indicates an ability to hinder IFN responses to viral infection and results in increased disease severity.^10^ In a recent study, type I IFN auto‐Abs without neutralizing activity in vitro were found frequently in patients admitted to the ICU (16%) regardless of SARS‐CoV‐2 infection; however, only auto‐Abs neutralizing type I IFNs were found in severe COVID‐19 patients admitted to the ICU and were associated with increased mortality.^10^ A similar finding was reported by Bastard et al.^9^ in which auto‐Abs that neutralize type I IFN were only found in severe COVID‐19 patients and were associated with a high mortality rate.^9^, ^10^ We further observed a high mortality rate in auto‐Abs positive patients with high neutralizing activity in vitro compared to patients with low neutralizing activity. These data suggest that the neutralization of at least one type I IFN might underlie the initial disease severity of MERS and may be associated with worse clinical outcomes.

The baseline characteristics of patients with or without type I IFN auto‐Abs were similar in terms of age, gender, body mass index (BMI), and comorbidities. It has been shown previously that auto‐Abs against type I IFNs significantly increased the COVID‐19 mortality rate at all ages.^29^ Although our cohort with type I IFN auto‐Abs were severely ill and admitted to the ICU at randomization, the mortality rate was similar to patients without type I IFN auto‐Abs. This could be due to the treatment intervention with lopinavir‐ritonavir and IFN‐β1b, which result in improved overall survival in the auto‐Abs positive patients because treatment with IFN‐β therapy has been shown to be effective in the treatment of patients with MERS.^24^ We evaluated the effect of the presence of type I IFN auto‐Abs and the treatment effect of the trial intervention (IFN‐β1b and lopinavir‐ritonavir) and found no statistically significant difference in the treatment effect between antibody positive and negative groups. However, this study is limited by its small cohort size, which restricted the study power. Future prospective work on tType I IFN auto‐Abs in MERS is needed.

In conclusion, auto‐Abs against at least one type I IFNs were common among hospitalized patients with MERS. Patients with type I IFN auto‐Abs were more likely to be critically ill. The presence of type I IFN auto‐Abs was not associated with clinical outcome or with a difference in the response to treatment with IFN‐β1b and lopinavir‐ritonavir.

## AUTHOR CONTRIBUTIONS

Faizah Alotaibi and Yaseen M. Arabi conceived and designed the study, analyzed the results, and wrote the manuscript. All other authors conducted the study, collected data, revised the manuscript, and approved the final version.

## CONFLICT OF INTEREST STATEMENT

YA provided nonpaid consultations on therapeutics for MERS for Gilead Sciences and SAB Biotherapeutics, and he is a board member of the International Severe Acute Respiratory and Emerging Infection Consortium (ISARIC). All other authors declare no financial or commercial conflict of interest.

### ETHICS STATEMENT

The study was approved by the IRB of the Ministry of National Guard Health Affairs (MNG‐HA), Riyadh, Saudi Arabia.

### PEER REVIEW

The peer review history for this article is available at https://publons.com/publon/10.1111/irv.13116.

## Supporting information


**Table S1:** Baseline characteristics and mortality of patients with MERS based on auto‐Abs neutralizing activity in vitro.
**Figure S1:** Forest plot of treatment effect with IFN‐β1b and lopinavir‐ritonavir among auto‐Abs positive and negative patients (p‐value for interaction = 0.49).
**Figure S2:** Kaplan‐Meier survival curve for (A) Auto‐Abs negative group and (B) Auto‐Abs positive group.

## Data Availability

The datasets generated and/or analyzed during the current study are available from the corresponding author upon reasonable request once all planned analyses have been completed and published or presented and after signing sharing agreement in accordance with the policies of KAIMRC for 3 years after the publication of this paper.
