# Supplementary material for: Type I interferon autoantibodies in hospitalized patients with Middle East respiratory syndrome and association with outcomes and treatment effect of interferon beta‐1b in MIRACLE clinical trial
Source: Influenza Other Respir Viruses. 2023 Mar 21;17(3):e13116. doi: 10.1111/irv.13116 (PMC10028524; doi:10.1111/irv.13116)
Supplement: Supplementary file 1 — Table S1: Baseline characteristics and mortality of patients with MERS based on auto‐Abs neutralizing activity in vitro. Figure S1: Forest plot of treatment effect with IFN‐β1b and lopinavir‐ritonavir among auto‐Abs positive and negative patients (p‐value for interaction = 0.49). Figure S2: Kaplan‐Meier survival curve for (A) Auto‐Abs negative group and (B) Auto‐Abs positive group. [file IRV-17-e13116-s001.docx]

**Supplementary file**

**Title: Type I Interferon autoantibodies in hospitalized patients with Middle East respiratory syndrome and association with outcomes and treatment effect of interferon beta‐1b in MIRACLE clinical trial**

**Contents**

**Collaborators**

**Supplementary tables:**

**Table S1:** Baseline characteristics and mortality of patients with MERS based on auto-Abs neutralizing activity in vitro.

**Supplementary figures**

**Figure S1:** Forest plot of treatment effect with IFN‐ β1b and lopinavir‐ritonavir among auto-Abs positive and negative patients (p for interaction = 0.49).

**Figure S2:** Kaplan-Meier survival curve for (A) Auto-Abs negative group and (B) Auto-Abs positive group.

| **Collaborators** |  |
| --- | --- |
| **King Abdullah International Medical Research Center** | Faizah **Alotaibi**  Naif **Alharbi**  Hanan H **Balkhy**  Majed **Al Jeraisy**  Ahmad M. **Deeb**  Jesna **Jose**  Badriah M **AlMutairi**  Abdulaziz **Al-Dawood**  Mashan L. **Abdullah**  Tlili **Barhoumi**  Mohammed W. **Alenazi** Abdulrahman **Almasood**  Yaseen M **Arabi** |
| **The Saudi Critical Care Trials Group** |  |
| King Saud bin Abdulaziz University for Health Sciences and King Abdullah International Medical Research Center, Riyadh, Saudi Arabia | Yaseen M. **Arabi**  Adel **Alothman**  Hanan H **Balkhy**  Abdulaziz **Al-Dawood**  Sameera **AlJohani**  Shmeylan **Al Harbi**  Suleiman **Kojan**  Majed **Al Jeraisy**  Naif Khalaf **Alharbi**  Ahmad M. **Deeb**  Badriah M **AlMutairi**  Jesna **Jose**  Mohammed **Al Mohaidib** Musharaf **Sadat**  Hala **Al Anizi**  Reggie **Dael**  Mohamed W. **Alenazi**  Haya A. **Aljami**  Ali **Alshehri** |
| Ministry of Health, Saudi Arabia | Abdullah M **Assiri**  Hani A. Aziz **Jokhdar**  Mohammad **AlMazroa**  Fawaz **Al-Rasheedi** |
| Prince Mohammed bin Abdulaziz Hospital, Riyadh, Saudi Arabia | Ayed Y. **Asiri**  Ziad A **Memish**  Sameeh S **Ghazal**  Sarah H **Alfaraj**  Mohammed **Alshaikh**  Dhaifallah Saud **Alotaibi**  Mostafa **Rajab**  Fatima Emieraiza P. **Isdung**  Chloe D. **Abinal**  Ruchil S. **Escobanez**  Carlos B. **landingin**  Samah **Badamas**  Norah Abdullatif **Hawsawi**  Hanan **Alanazi**  Anwar Ali **Mohammed**  Hail **Al Nono**  Ali Othman **Alkahlaf**  Fahad **Al Daeaji**  Ahmed **Madi**  Abdulrahaman **Idrees** |
| King Fahad Medical City, Riyadh, Saudi Arabia | Abdullah **Al Motairi**  Mushira **Al Enani**  Alaa **Alqurashi**  Fatimah **Alenezi**  Nada **Alkhani** |
| Prince Sultan Military Medical City, Riyadh, Saudi Arabia | Yasser **Mandourah**  Ghaleb A. **AlMekhlafi**  Nisreen Murad **Sherbeeni**  Fatehi Elnour **Elzein**  Shatha Anwar **Al Samarrai**  Rima E **Mahamed**  Abdulrauf Ahmed **Malibary**  Bander **Al Anezi**  Ma. Raylin **Cubio**  Melvin **Salunga**  Shatha Moayad **Awad**  Maha E. **Aljuhani**  Ghena **Jaber**  Adnan **Alghamedi**  Osama **Elfaki**  Najlaa **Almutairi** |
| King Saud Medical City, Riyadh, Saudi Arabia | Abdulrahman **AlHarthy**  Mohammed **AlSulaiman**  Ahmed **Mady**  Basheer **Abdulrhman** Tasmyia **Asaad** Gultakin **Bakirova**  Amany **Albraiky** Hamad **AlShahrany**  Huda **Mhawish**  Alva **Alcazar** |
| King Faisal Specialist Hospital & Research Center, Riyadh, Saudi Arabia | Khalid **Maghrabi** |
| King Abdulaziz Medical City, Jeddah, Saudi Arabia | Fahad **Al-Hameed**  Asim **Alsaedi**  Ohoud **Aloraabi**  Jalal **Refai**  Pansy **Elsamadisi**  Medhat S **Hendy**  Sara AbuBaker **Basher** |
| King Abdullah Medical Complex, Jeddah, Saudi Arabia | Mohamed Hatem A **Azzam**  Muhammed **Abduldhaher**  Wael **Bajhamoum**  Hala Ibrahim **Alnazawi**  Mohammad Nassar **Almadani**  Mohannad Saud **Alnefaie** |
| King Abdulaziz Hospital-Alahsa, Saudi Arabia | Jamal **Chalabi**  Yusri **Taha**  Javed **Memon**  Shahinaz **Bashir**  Ibraheem **Al-Dossary**  Saleh **Al Mekhloof** |
| King Fahad Hospital, Al-Madinah Al-Monawarah, Saudi Arabia | Ayman **Kharaba**  Ahmad **Al Jabri** |
| Ohoud Hospital, Al-Madinah Al-Monawarah, Saudi Arabia | Ayman **Kharaba**  Magdy **Farid**  Alawi **Alaidarous**  Wael **Alseraihi**  Husam **Shahada**  Jinish **Shimi** |
| Aseer Central Hospital, Abha, Saudi Arabia | Ali **Al Bshabshe**  Abdelmoniem **Al Bahar**  Wafa **Qadri**  Bensi **Mathew**  Ahmad Mushabab **Assiri**  Ali **Alhusin**  Nora **Assiri** |
| King Faisal Medical Complex, Taif, Saudi Arabia | Hanadi Mohamed Ahmed **Ouali**  Lamya **Al Zubaidi**  Rhea Mae **Gesulga**  Badr Ali **Al Harthy**  Abed Suryeh **Algothemi**  Rinu Mary Raju **Philip**  Rajani **Rajan** |
| Dammam Medical Complex, Dammam, Saudi Arabia | Shahab **Alsuliman**  Hajer **Aldossery**  Mohammed **Alnabi** |
| King Fahad Hospital, Hofuf, Saudi Arabi | Mahmoud **Albagshi** |
| King Khalid Hospital, Najran, Saudi Arabia | Abdulhadi Mohmmed **Bin Eshaq**  Abduelbagi D A **Altayb**  Ezaldeen H **Omer**  Salem Saleh **AlQirad**  Fatimah Awaadh **Balhareth**  Sutharani **Esakkimuthu**  Jubinamol **Chacko**  Maryjoy **Arquiza**  Dhanyamol C.**Babu** |
| National Institute of Allergy and Infectious Diseases (NIAID), National Institutes of Health (NIH), Bethesda, MD, USA | Lindsey B. **Rosen**  Steven M. **Holland** |

**Table S1:** Baseline characteristics and mortality of patients with MERS based on auto-Abs neutralizing activity in vitro. The patients with auto-Abs that were positive for neutralizing activity in figure 3, were divided into low and high neutralizing activity in which “low” represents samples with less than 50% of neutralization and “high” represents samples with more than 50% of neutralization.

| Variable | Patients with auto-Abs neutralizing in vitro  (N = 6) | Patients with auto-Abs with low neutralizing in vitro  (N = 3) |
| --- | --- | --- |
| Age, years, median (IQR) | 60.5 (44 – 73) | 50.3 (23 – 67) |
| Gender, Male, no. (%) | (5/6) 83.3% | (2/3) 66.6% |
| Mortality, no. (%) | (5/6) 83.3% | (1/3) 33.3% |

**Figure S1:** Forest plot of treatment effect with IFN‐ β1b and lopinavir‐ritonavir among auto-Abs positive and negative patients (p for interaction = 0.49).


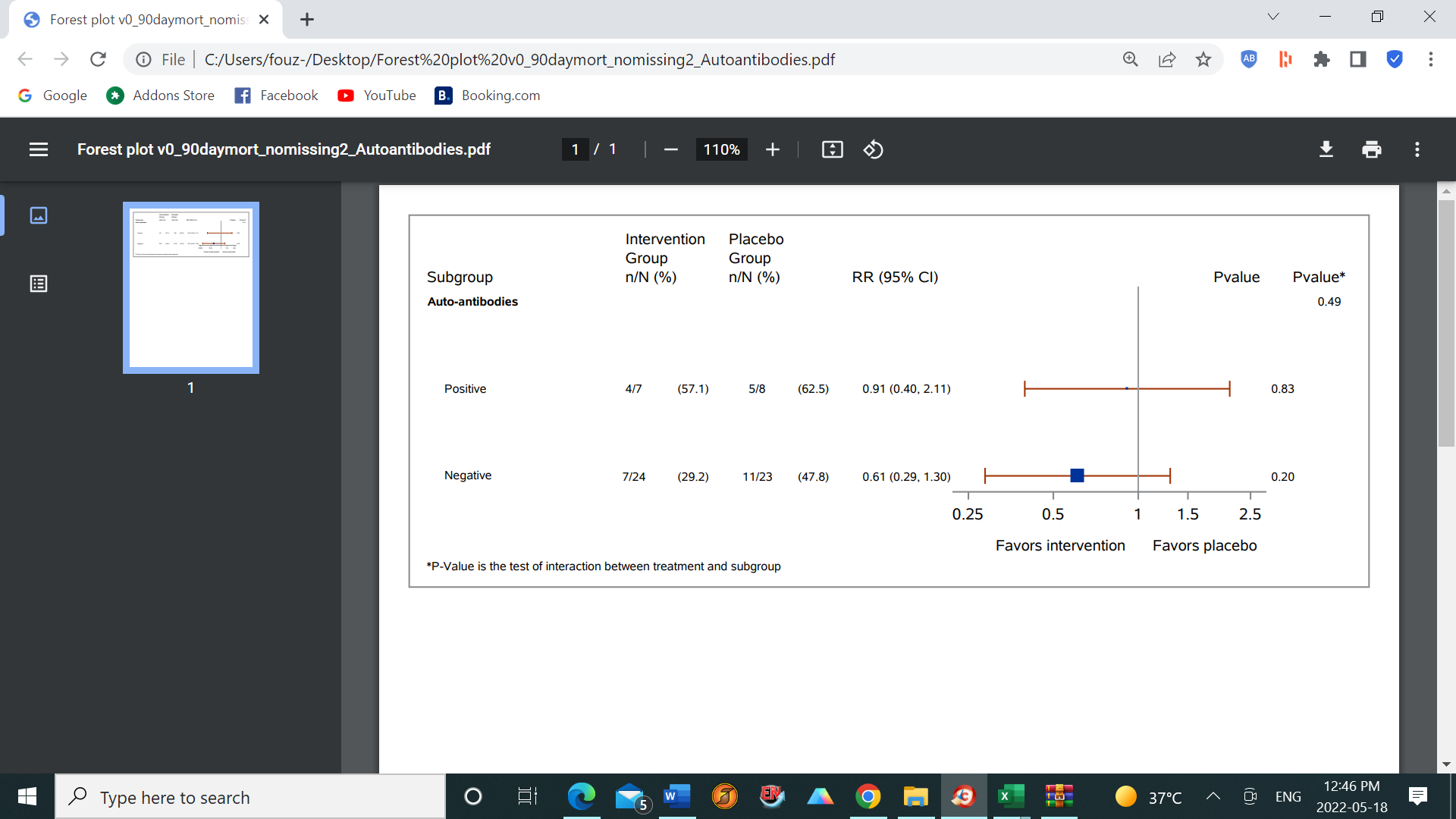


**Figure S2:** Kaplan-Meier survival curve for (A) Auto-Abs negative group and (B) Auto-Abs positive group.

| **Panel A:** Auto-Abs negative group |
| --- |
| **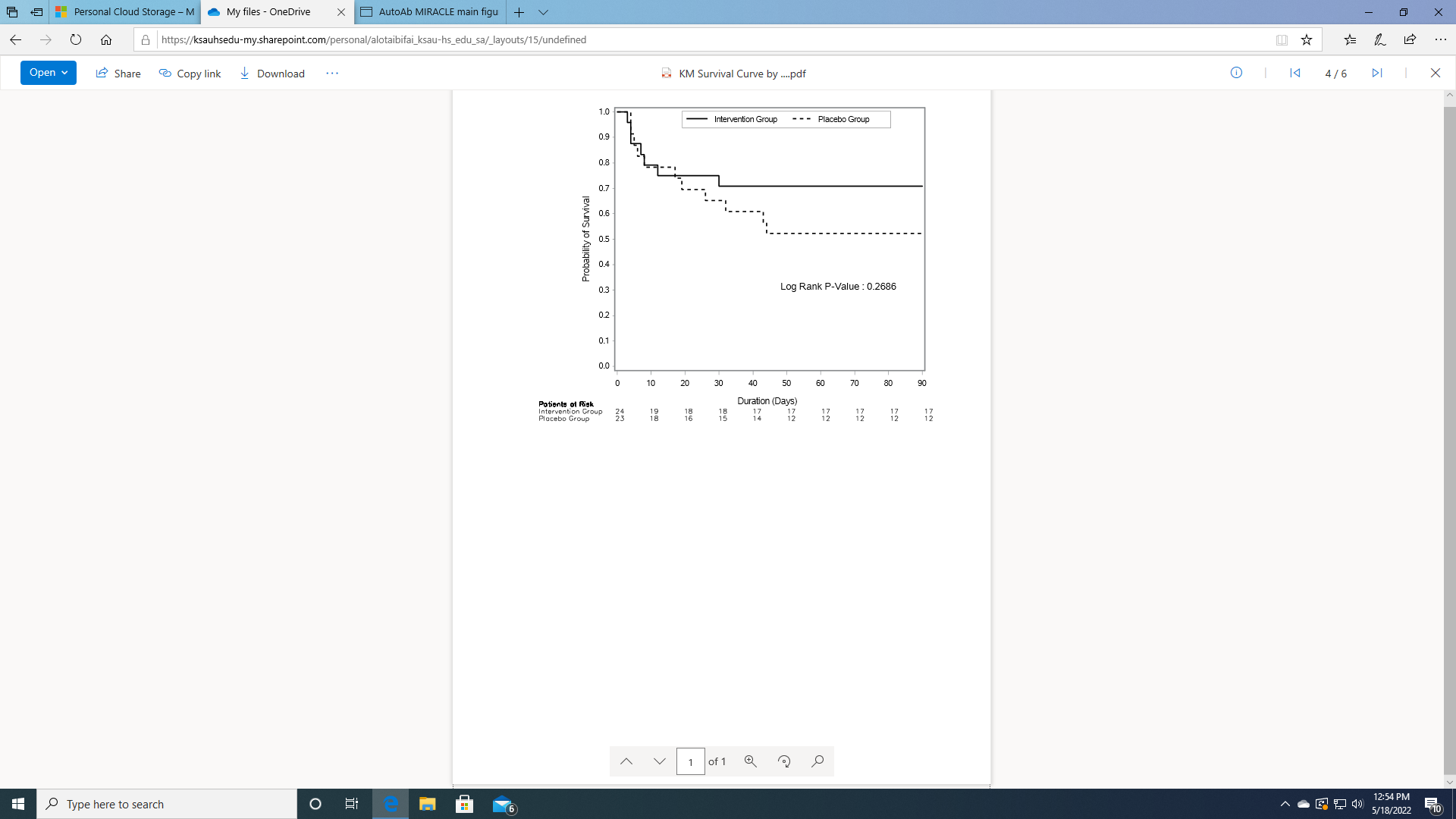** |
| **Panel B:** Auto-Abs positive group |
| **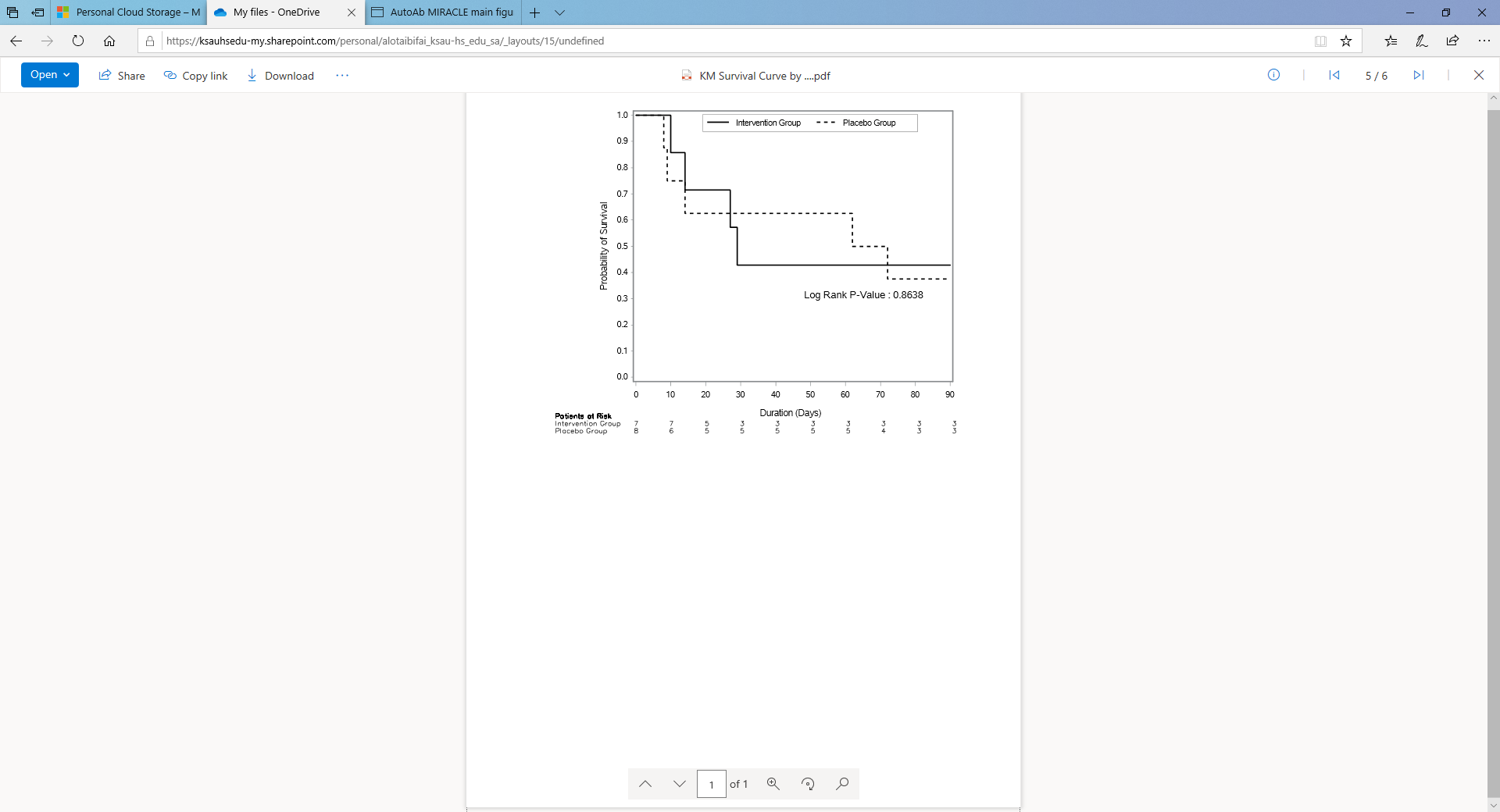** |
